# Immunization of mice with soluble lysate of interferon gamma expressing *Plasmodium berghei* ANKA induces high IFN-γ production

**DOI:** 10.1186/s40794-017-0053-1

**Published:** 2017-06-06

**Authors:** Ebenezer Taylor, Faith Onditi, Naomi Maina, Hastings Ozwara

**Affiliations:** 1 Department of Molecular Biology and Biotechnology, Pan African University, Institute for Basic Sciences, Technology and Innovation (PAUSTI), P.O. Box 6200-00200, Nairobi, Kenya; 2 Department of Biochemistry, School of Biomedical sciences, Jomo Kenyatta University of Agriculture and Technology (JKUAT), P.O. Box 62000-00200, Nairobi, Kenya; 3 Department of Tropical and Infectious Diseases, Institute of Primate Research (IPR), P.O. Box 24481-00502, Karen, Nairobi, Kenya

**Keywords:** Immunization, Interferon gamma, Malaria, *Plasmodium berghei*, Mouse IFN-γ expressing PbA

## Abstract

**Background:**

Efforts in search of lasting malaria vaccine have led to the development of transgenic rodent malaria parasites. As a result, wild type *Plasmodium berghei* ANKA (WTPbA) has recently been transformed to express mouse interferon gamma (mIFN-γ). The immunomodulatory effect of this transgenic parasite on WTPbA infection has been demonstrated. However, the protective immune responses after repeated immunization with soluble lysate of this parasite has not been investigated.

**Methods:**

Soluble lysate of transgenic PbA (TPbA) was prepared and concentration of IFN-γ in lysate determined by ELISA. Four groups of 20 BALB/c mice each (two treatment groups and two control groups) were setup. Treatment Groups 1 and 2 were primed (at day 0) with lysate of TPbA containing 75 pg/ml IFN-γ and live TPbA parasites respectively. Infection in Group 2 mice was cured with Coartem™ at 450 mg/kg for 3 days. At day 14 post-priming, both groups were boosted twice at day 14 and day 28 with lysate of TPbA containing 75 pg/ml IFN-γ and 35 pg/ml IFN-γ respectively. Blood and spleen samples were collected at day 0, day 14, day 21 and day 28 for preparation of serum and cell cultures respectively. Serum IgG and cytokines (TNF-α and IFN-γ) levels in culture supernatant were measred by ELISA.Survivorship and parasitemia were daily monitored for 21 days. Data were statistically analyzed using ANOVA student’s *t* test. A *p* value of <0.05 was considered significant.

**Results:**

At day 28 post-priming, IFN-γ production in Group 1 was tenfold higher than in RBC control group (*p* = 0.070) There was significant difference in IFN-γ production among the groups at day 28 (*p* < 0.0001). TNF-α production in Group 1 mice increased fourfold in Group 2 mice from day 14 to day 28 post-immunization (*p* = 0.0005). There was no significant effect on serum IgG production. Mice in treatment groups survived 5 to 4 days longer compared to non-immunized group.

**Conclusion:**

The study has demonstrated that, repeated immunization with soluble lysate of TPbA induces Th 1 response leading to increased IFN-γ and TNF-γ production.

## Background

Over the years, efforts have been made to reduce the burden of malaria globally [1]. In spite of these efforts, the disease continues to take a significant toll on developing economies [2]. According to a recent report by the World Health Organization (WHO), 200 million cases of malaria leading to over 400, 000 deaths were recorded in 2015 [1]. Majority of these cases (88%) and deaths (91%) occurred in the sub-Saharan Africa. The fight against the disease by way of currently existing control measures is further compounded by the emergence of drug resistance parasites [3] and insecticide resistance vectors [4]. Unfortunately, a licensed vaccine to avert the disease in all age groups is currently unavailable [5].

A vaccine against *Plasmodium* is regarded as the most effective way to control malaria and the discovery of one would contribute towards malaria prevention especially in endemic areas. The path to a successful malaria vaccine development is however inundated with many challenges such as identification of protective immune correlates [6]. During *Plasmodium* infection, both innate and adaptive immune cells are induced to produce pro-inflammatory cytokines via different cellular mechanisms [7, 8]. Evaluation of these cytokines in many studies have revealed that, IFN-γ production plays a critical role in mediating protective immunity against both the pre-erythrocytic and blood-stage parasites [9–11]. Therefore, any effort towards developing a vaccine aimed at or producing IFN-γ responses would be highly desirable.

With the advancement in transfection technology for malaria parasites, mIFN-γ expressing PbA has recently been generated in the malaria lab at Institute of Primate Research (IPR) [12]. This transgenic PbA (TPbA) has been shown to express bioactive host IFN-γ that immunomodulate immune responses against malaria parasites [12]. However, the effect of repeated immunization on Th1 and B-cell responses leading to production of protective immunity has not yet been investigated. The study was therefore designed to induce protective immunity against wild type blood-stage *Plasmodium berghei* (WT PbA) by priming-boosting immunization strategy.

## Methods

### Experimental animals and parasites

For all experiments, 6 to 10 weeks old male and female BALB/c mice were used. The mice were bred and maintained at the pathogen-free facility of the Institute of Primate Research (IPR) on standard pellet diet and water ad libitum in a 12-h alternating light–dark cycle. The transgenic *P. berghei* ANKA and wild type *P. berghei* ANKA (WtPbA) parasites obtained from IPR were used in this study. The generation and selection of TPbA parasites has been described previously by [12].

### In vivo propagation and maintenance of TPbA parasites

A vial of TPbA was retrieved from liquid nitrogen storage, thawed in water bath at 37 °C and inoculated into two mice. Upon detection of parasites by microscopy, mice were introduced to drinking water containing pyrimethamine (0.07 mg/ml) for selection of transformed parasites [13]. Transformed parasites were then maintained in BALB/c mice by serial passages (at 4 to 6% parasitemia). Passages were done by intraperitoneal (i.p) injection of mice with 2 × 10^6^ parasitized red blood cells (pRBCs) and parasitemia monitored by microscopy [14]. The effectiveness of Pyrimethamine used was pre-tested in vivo and compared with wild type non-resistant *Pb*A. Freezing of the pRBC in liquid nitrogen was done as previously described [15].

### In vitro propagation and preparation of TPbA soluble lysate

Ten mice were i.p inoculated with 2 × 10^6^ parasites and treated with Pyrimethamine as described above [13] . At 6 to 10% parasitemia, mice were euthanized in a CO_2_chamber and blood harvested by cardiac puncture and washed thrice with incomplete RPMI 1640 medium [16]. The pellet was resuspended in complete RPMI 1640 medium [incomplete medium supplemented with 25% fetal bovine serum (FBS)] at 5% hematocrit and aseptically transferred into four T-25 culture flasks. The culture was then inoculated with pyrimethamine at 100 nmol/l [17], flushed with gas mixture (5% CO_2_, 5% O_2_, 90% N_2_) and incubated overnight for 18 h at 36.5 °C. After the 18 h, the culture was washed once at 200 g for 10 min at 24 °C and supernatants collected (stored at -80 °C). The pellet was resuspended at 20% hematocrit and incubated for additional 9 h (i.e making a total of 27 h). After the 9 h, the culture was sonicated briefly on ice for 5 min at 30s pulse and 1 min rest using 2–3 amplitude microns for rupturing of matured schizonts. The parasite lysate was then centrifuged at 200 g for 10 min at 4 °C and supernatant filter-sterilized using 0.45 μm-pore syringe filters. The soluble lysate was aliquoted and stored at -80 °C for measurement of IFN-γ concentration by enzyme linked immunosorbent assay (ELISA) and immunization later.

### Immunization, challenge infection, and sample collection and processing

Eighty BALB/c mice were randomly assigned into four groups of 20 mice each (two treatment groups and two control groups). The treatment groups were Group 1 and Group 2 whereas Group 3 and Group 4 constituted the control groups. Mice in Group 1 were primed by i.v injection with 200 μl of TPbA lysate containing 75 pg/ml. At day 14 post-priming, the mice were boosted twice at 7 days interval. For Group 2, the mice were primed with live TPbA parasites (2 × 10^6^ in 200 μl RPMI)and at 4 to 6% parasitemia, mice were treated orally with Coartem™ (Novartis, Basel, Switzerland) at 450 mg/kg body weight for three consecutive days. At day 14 pos-priming, the mice were boosted twice with TPbA lysate containing 35 pg/ml IFN-γ (200 μl) at 7 days interval. Soluble lysate of plain RBC (Group 3) and WT PbA parasite and its lysate (Group 4) were used in the control groups. Seven days after the last boost (at day 28), mice (*N* = 5/4) from each group were then challenged with 2 × 10^6^ WT PbA parasites (200 μl in PBS). Naive (non-immunized) control group was introduced at the point of challenge. Parasitemia and survivorship were then monitored from day 1 post-challenged infection for 21 days.

Baseline sampling (N=5) was done at the beginning of the experiment (Day 0). Afterwards, mice (N=5) were euthanized with CO_2_ at three time points (day 14, day 21, and day 28). Blood was then collected by cardiac puncture for serum isolation. The spleen of each mouse was also aseptically removed and processed as described previously by [18]. The cells were enumerated by trypan blue exclusion method and plated at 3 x 105 cells/ 1mL complete medium in a 48-well culture plate in duplicates in the presence or absence of 1µg concanavalin A (ConA) [19]. The plates were incubated for 72 hours at 37^o^C in a humidified incubator supplied with 5% CO2. After incubation, culture supernatant was collected and stored at -80^o^C. 

### Measurement of serum IgG and cytokines (IFN-γ and TNF-α)

Measurement of Total serum IgG levels and cytokines levels in culture supernatants was done using commercial ELISA kits (mouse IgG, IFN-γ and TNF-α) according to manufacturer’s instructions (Mabtech, Nacka Strand, Sweden). The limit of detection of the assays were 0.1 ng/ml, 2 pg/ml and 6 pg/ml for IgG, IFN-γ and TNF-α respectively. Cytokine levels were expressed as amount produced under experimental conditions after subtracting cytokines released by control cultures containing media alone.

### Statistical analysis

Data were first entered and managed in Microsoft Excel and subsequently analyzed using GraphPad Prism® Version 5.01 software (La Jolla, CA, USA). Comparison of immune responses was done using ANOVA and student’s *t* test. A *p* value of <0.05 was considered significant.

## Results

### Concentration of mouse IFN-γ in soluble parasites lysate

Mouse IFN-γ was detectable in both control (RBC and wild type PbA) and TPbA cultures. IFN-γ concentration was 51.87 pg/ml in WTPbA culture and 43.83 pg/ml in TPA culture. After 27 h with sonication, IFN-γ concentration in TPbA soluble lysate was about elevenfold higher compared to WTPbA soluble lysate (Table 1).

**Table 1:** Concentration of mIFN-γ in Soluble Lysate

Culture Type	18 h	27 h + sonication
Transgenic *Pb*A (TPbA)	43.83 pg/ml	714.83 pg/ml
Wild type *Pb*A (WTPbA)	51.87 pg/ml	63.32 pg/ml
Uninfected RBC	<20 pg/ml	<20 pg/ml

Immunization of mice with mIFN-γ expressing PbA soluble lysate has significant effect on cytokine level production

### Immunization of mice with mIFN-γ expressing PbA soluble lysate has significant effect on cytokine production

**Fig. 1:**
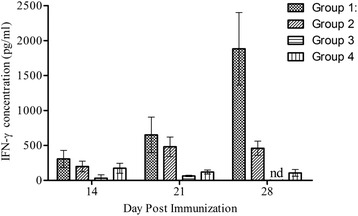
Effect of soluble lysate of mIFN-γ expressing PbA on IFN-γ production in mice. Error bars represent mean ± standard error of mean. Group 1: Primed with TPbA lysate (75pg/ml IFN-γ), boosted with TPbA lysate (75pg/ml IFN-γ); Group 2: Primed with live TPbA parasites, boosted with TPbA lysate (35pg/ml IFN-γ); Group 3; Primed with plain RBC lysate, boosted with plain RBC lysate (Plain RBC Control); Group 4: Primed with live WTPbA parasites, boosted with WTPbA lysate (Wild Type control); nd: value below baseline levels

There was significant effect on Th1 immune responses after mIFN-γ-containing soluble lysate immunization. At day 14 post-priming, IFN-γ production in Group 1 mice was tenfold higher compared to Group 3 mice (*p* = 0.071; Mean ± SEM: 306.6 ± 122.3 pg/ml; 30.66 ± 50.40 pg/ml). Similarly, IFN-γ production in Group 1 mice was higher than production in Group 2 mice (*p* = 0.476; Mean ± SEM: 306.6 ± 122.3 pg/ml; 198.7 ± 76.3 pg/ml). There was significant difference in IFN-γ among the groups at day 21 (*p* = 0.031) and 28 (*p* < 0.0001). Compared to Group 2, IFN-γ production was significantly higher in Group 1 (*p* = 0.032). There was significant increase in production from day 14 to day 28 in Group 1 mice (*p* = 0.021; Mean ± SEM: 306.6 ± 122.3 pg/ml; 1882 ± 519.2 pg/ml). IFN-γ production in Group 2 mice also doubled at day 28 (*p* = 0.070; Mean ± SEM: 198.7 ± 76.34 pg/ml; 459.8 ± 101.0 pg/ml) (Fig. 1 **).**

**Fig. 2:**
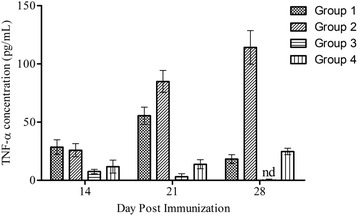
Effect of immunization on TNF-α responses in mice. Error bars represent mean ± SEM. Group 1: Primed with TPbA lysate (75pg/ml IFN-γ), boosted with TPbA lysate (75pg/ml IFN-γ); Group 2: Primed with live TPbA parasites, boosted with TPbA lysate (35pg/ml IFN-γ); Group 3; Primed with plain RBC lysate, boosted with plain RBC lysate (Plain RBC Control); Group 4: Primed with live WTPbA parasites, boosted with WTPbA lysate (Wild Type control); nd: data below baseline levels

Immunization of mice with soluble lysate of TPbA resulted in induction of low grade TNF-α production (Fig. 2 **).** At day 14 post-priming, TNF-α production was significantly different among the groups (*p* = 0.02). Compared to Group 3, TNF-α production in Group 1 was fourfold higher (*p* = 0.01; Mean ± SEM: 28.52 ± 6.232 pg/ml; 7.530 ± 1.909 pg/ml). There was no significant difference in TNF-α production between Group 2 and Group 3 (*p* = 0.11; Mean ± SEM: 11.77 ± 5.576 pg/ml; 25.91 ± 5.684 pg/ml). TNF-α in Group 1 mice increased from day 14 to day 28 post-immunization (*p* = 0.19; Mean ± SEM: 28.52 ± 6.232 pg/ml; 18.23 ± 3.738 pg/ml). In contrast, TNF-α production increased fourfold in Group 2 mice from day 14 to day 28 post-immunization (*p* = 0.0005; Mean ± SEM: 25.91 ± 5.684 pg/ml; 114.2 ± 14.45 pg/ml). At day 28, TNF-α production was significantly higher in Group 2 mice than in Group 1 mice (*p* = 0.0002; Mean ± SEM: 114.2 ± 14.45 pg/ml; 18.23 ± 3.738 pg/ml). Compared to Group 4, at day 28 post-immunization, TNF-α production in Group 2 mice was significantly higher (*p* = 0.0003; 114.2 ± 14.45 pg/ml; 24.79 ± 2.798 pg/ml). These results show that, priming and boosting of mice with mIFN-γ-containing soluble lysate are more potent in inducing IFN-γ production than priming with live TPbA parasite and boosting with mIFN-γ-containing soluble lysate. Similarly, the results also demonstrate that, priming of mice with live TPbA parasites induces TNF-α production better compared to priming with mIFN-y-containing soluble lysate.

### Immunization of mice with TPbA lysate has no significant effect on IgG levels

**Fig. 3:**
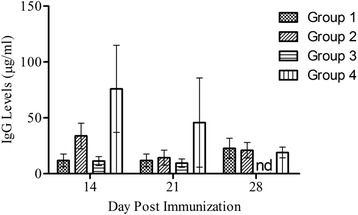
Total Serum IgG response in immunized mice. Error bars represent mean ± SEM. Group 1: Primed with TPbA lysate (75pg/ml IFN-γ), boosted with TPbA lysate (75pg/ml IFN-γ); Group 2: Primed with live TPbA parasites, boosted with TPbA lysate (35pg/ml IFN-γ); Group 3; Primed with plain RBC lysate, boosted with plain RBC lysate (Plain RBC Control); Group 4: Primed with live WTPbA parasites, boosted with WTPbA lysate (Wild Type control); nd: value below baseline levels

The effect of immunization on total serum IgG production on mice was also analyzed (Fig. 3). At day 14 post-priming, IgG production in Group 1 (Mean ± SEM: 11.86 ± 5.734 μg/ml) and Group 2 (33.79 ± 11.44 μg/ml) mice was lower compared to Group 4 (Mean ± SEM: 75.94 ± 38.98 μg/ml) mice (*p* = 0.128 and *p* = 0.575 respectively). Similarly, there was no significant difference in IgG production between the groups at day 14 and day 28 (*p* > 0.05). These results suggest that, immunization of mice with mIFN-γ-containing soluble lysate had no significant effect on B-cell responses. However, higher B-cell responses may be induced upon repeated immunization,

### Immunization of mice with TPbA lysate induces protection against blood-stage infection of WT PbA

**Fig. 4:**
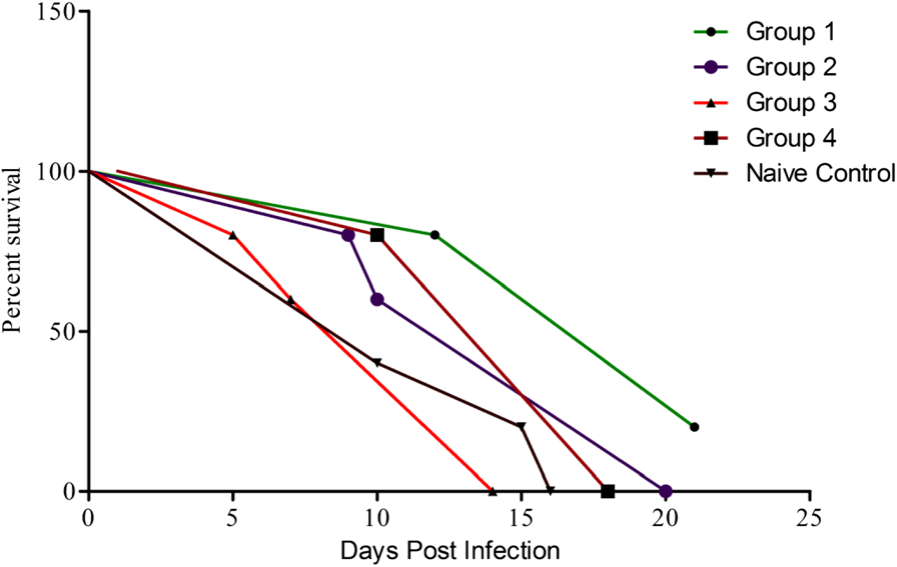
Survival curve of immunized and non-immunized mice. Group 1: Primed with TPbA lysate (75pg/ml IFN-γ), boosted with TPbA lysate (75pg/ml IFN-γ); Group 2: Primed with live TPbA parasites, boosted with TPbA lysate (35pg/ml IFN-γ); Group 3; Primed with plain RBC lysate, boosted with plain RBC lysate (Plain RBC Control); Group 4: Primed with live WTPbA parasites, boosted with WTPbA lysate (Wild Type control)

Parasitemia patency delayed 4 days longer in Group 1mice compared to non-immunized control group. Also, parasitemia patency in the non-immunized control group was 3 days shorter than in Group 2 mice. The parasite growth rate in Group 1 was significantly reduced with 97.20% parasitemia suppression on day 7 relative to the non-immunized control group. The mean patent parasitemia in Groups 1 and 2 was also lower compared to the control groups (Table 2). Mice in Group 1 and Group 2 respectively, survived challenge infection 5 days and 4 days longer compared to non-immunized control group (Fig. 4).

**Table 2:** Protection of BALB/c mice immunized with mIFN-γ against wild type PbA challenge infection

Group	Pre-Patency Period (days)^a^	Positive mice/Challenge (% Protected)	Mean Patent Parasitemia (%)
Group 1	7	2/4^b^ (50%)	0.18
Group 2^c^	6	2/4^b^ (50%)	0.46
Group 3	3	2/5 (60%)	0.5
Group 4	3	2/4^b^ (50%)	2.44
Naive Control	3	2/5 (50%)	1.01

Group 1: Primed and boosted with TPbA lysate (75 pg/ml IFN-γ), Group 2: Primed with live TPbA, boosted with TPbA lysate (75 pg/ml IFN-γ); Group 3: Plain RBC control; Group 4: WT PbA control

^a^The period of detecting parasites in blood after challenge infection

^b^One mouse died unexpectedly before challenge infection. Death not due to immunization

^c^Mice were primed with transfected PbA and boosted twice with 35 pg/ml mIFN-γ containing soluble lysate

## Discussion

In this present study, immune responses to host interferon gamma expressing PbA were assessed. Immunization with soluble lysate induced high IFN-γ production and low grade TNF-α production by Th1 cells. The production of these cytokines were observed to increase with boosting. IFN-γ production but not TNF-α levels were higher in mice immunized with only TPbA soluble lysate (Group 1) compared to mice immunized with live TPbA plus soluble lysate (Group 2). Immunization in both groups had no significant effect on serum IgG levels compared to WT PbA control. It was observed that, Group 1 and Group 2 mice survived infection 5 to 4 days longer than the WT PbA control animals. These results suggest that repeated immunization is required to induce substantial levels of Th1 responses leading to enhanced protection.

In this study, concentration of IFN-γ in overnight (18-h) cultures was sixteenfold lower compared to 27-h sonicated culture. When compared to the concentration measured in previous study, IFN-γ level detected in 27-h sonicated was about fourfold higher [12]. This may probably be due to the mechanical rupture of mature schizonts leading to the release of proteins expressed by the parasite and which are usually trapped in the cytoplasm of the erythrocytes [20].

Significant levels of TNF-α and IFN-γ were detected in spleen cells of mice both treatment groups after first boost. It has been reported that, IFN-γ affects monocytes by shifting its differentiation from dendretic cells to macrophages [21]. Similarly, exposure of macrophages to IFN-γ has been shown to enhance secretion of other cytokines (TNF-α and IL-12) and promotion of Th1 cell development [22]. Thus, the gradual increase of TNF-α and IFN-γ levels observed upon boosting is due to priming of Th1 lymphocytes and activated macrophages [23]. The IgG produced may be as a result of B-cells activation by CD4^+^ produced IFN-γ [24, 25].

The delayed parasitemia patency coupled with prolonged parasitemia suppression and survival time in the treatment groups may be due to the high IFN-γ and TNF-α levels present prior to challenge infection. This observation has been reported in previous studies where clearance of blood stage infection coincides with high IFN-γ levels [23, 26, 27]. Other studies have equally confirmed the involvement of TNF-α in *Plasmodium* clearance [24], [25]. TNF-α act to induce parasite clearance by activating of other cells such as macrophages which leads to the release of nitric oxide (NO) [28]. Release of NO leads to killing of parasites [29].

Despite the high IgG levels in WTPbA control (Group 4) compared to Group 1, pre-patency period and survival time were shorter. This suggests that, IgG alone is not effective in promoting prolong parasite suppression and enhanced survival and this is in agreement with earlier study [30, 31].

In this study, priming and boosting of mice with soluble lysate (Group 1) induced better protective immune responses compared with priming with the cytokine expressing parasites and boosting with the soluble lysate (Group 2). This observation could possibly be due to differences in signalling path of inducing Th 1 immune responses [32].

Both IFN-γ and TNF-α are also involved immune mediation during human malaria [23–27]. Thus, development of transgenic human malaria parasite expressing host IFN-γ has desirable implications in blood-stage malaria vaccine development strategies.

## Conclusion

In this study, mIFN-γ expressing PbA parasite recently generated in the malaria lab of IPR was used. Soluble lysate containing mIFN-γ was prepared and used for the immunization. The study has demonstrated that, immunization with soluble lysate is potent in inducing high IFN-γ production and repeated immunization increased further the responses. Moreover, the study has revealed that priming of mice with the transfected parasites induces better TNF-α responses than soluble lysate containing IFN-γ. Thus combining the live transgenic parasites with the soluble lysate in a priming-boosting manner would be highly desirable. Further studies are required to determine whether parasite expressed IFN-γ is secreted out by the erythrocyte, or released upon schizont rupture.

## Abbreviations

ConA: Concanavalin A
ELISA: Enzyme-linked immunosorbent assay
FBS: Fetal bovine serum
i.p: Intraperitoneal
i.v: Intravenous
IFN-γ: Interferon gamma
IgG: Immunoglobulin G
IPR: Institute of Primate Research
PbA: *Plasmodium berghei* ANKA
TNF-α: Tumour necrosis factor alpha
TPbA: Transgenic PbA
WTPbA: Wild type PbA
